# Predictors of Cardiac Resynchronization Therapy Response: The Pivotal Role of Electrocardiogram

**DOI:** 10.1155/2013/837086

**Published:** 2013-03-20

**Authors:** Yahya S. Al Hebaishi, Halia Z. Al Shehri, Abdulrahman M. Al Moghairi

**Affiliations:** ^1^Adult Cardiology Department, Prince Sultan Cardiac Centre (PSCC), Prince Sultan Military Medical City, P.O. Box 27656, Riyadh 11427, Saudi Arabia; ^2^Adult Cardiology Department, Prince Salman Heart Center, King Fahad Medical City, P.O. Box 59046, Riyadh 11525, Saudi Arabia

## Abstract

Heart failure affects millions of patients all over the world, and its treatment is a major clinical challenge. Cardiac dyssynchrony is common among patients with advanced heart failure. Resynchronization therapy is a major advancement in heart failure management, but unfortunately not all patients respond to this therapy. Hence, many diagnostic tests have been used to predict the response and prognosis after cardiac resynchronization therapy. In this paper we summarize the usefulness of different diagnostic modalities with special emphasis on the role of surface electrocardiogram as a major predictor of response to cardiac resynchronization therapy.

## 1. Introduction

Heart failure is estimated to affect more than 23 million people worldwide with an approximately 2 million new cases diagnosed annually [1]. In the United States it is estimated that 5.1 million people have HF [2]. The incidence of heart failure increases with age, with approximately 10 in every 1,000 at age above 65 years being affected [2, 3]. Left bundle branch block (LBBB) and wide QRS complex are surrogates of left ventricular dyssynchrony that are commonly found in heart failure patients, and their presences associated with increased mortality [4–6]. In addition to medical therapy, implantable device therapy has become a standard therapy for refractory heart failure. Cardiac resynchronization therapy (CRT) has been shown to improve symptoms, quality of life, and survival and to enhance reverse remodeling in appropriately selected patients [7–9]. The efficacy of such therapy was demonstrated in patients with moderate and severe heart failure and more recently patient with mild heart failure symptoms [7–13]. Albeit the clinical response to CRT is evident in the majority of case, the lack of response still seen in approximately one-third of patients [7]. In this paper we discuss the potential value of different imaging modalities and ECG parameters in predicting CRT response.

## 2. Patient's Selection for CRT: Is There Still a Role for Echo and Other Imaging Modalities?

Correction of left ventricular (LV) dyssynchrony is thought to be the main therapeutic effect of CRT. In the past decade several imaging techniques were used to quantify mechanical dyssynchrony and predict CRT response; these imaging techniques include M-mode echocardiography, Tissue Doppler imaging (TDI), Strain imaging, 3-dimensional echocardiography, magnetic resonance imaging, and nuclear cardiology. In addition to the technical difficulty and increased cost associated with the use of these imaging techniques, the accuracy of such modalities in predicting CRT is questionable.

Multiple echocardiographic parameters had been shown to correlate with the response to CRT in several trials; however, the PROSPECT, large, multicenter, and prospective study, of 498 patients demonstrated that the tested 12 different echocardiographic dyssynchrony measures were unable to distinguish responders from nonresponders to a degree that may influence clinical decision [9, 14–16]. 

Real-time 3-dimensional echocardiography (RT3DE) is an emerging technique for left ventricular (LV) dyssynchrony assessment. The advantage of RT3DE is its ability to provide simultaneous information of the global LV contractility [17]. In a series of 57 consecutive heart failure patients scheduled for CRT, Marsan et al. evaluated the systolic dyssynchrony index (SDI) obtained by RT3DE. SDI cutoff value of 6.4% yielded a sensitivity of 88% and specificity of 85% to predict response to CRT [18]. In another study of sixty heart failure patients, triple plane TDI was able to predict six months clinical response and reverse LV remodeling after CRT implantation with a sensitivity of 89% and specificity of 82% [19]. Despite the promising early studies these techniques have their own limitations and need further validation.

**Figure 1:**
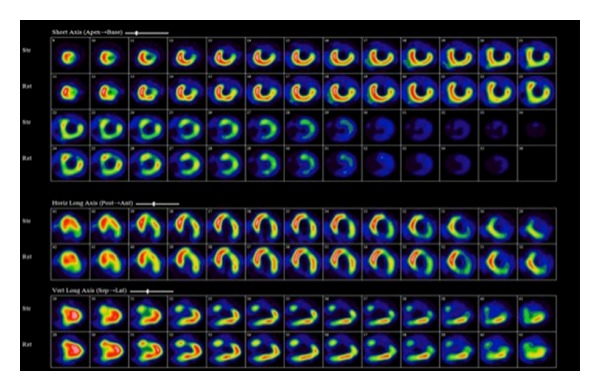
Baseline Tc-99m SPECT myocardial perfusions scan from CRT candidate demonstrating a fixed perfusion defect involving anterior and anterolateral wall consistent with transmural scar. Intraoperative testing demonstrated a high pacing threshold at anterolateral LV lead position; excellent pacing threshold was obtained from a posterolateral coronary sinus branch.

Nuclear imaging with single photon emission computed tomography (SPECT) and magnetic resonance imaging (MRI) are another modalities, which have been used in the assessment of LV mechanical dyssynchrony. Additional advantage of both techniques is their ability to assess the presence and location of LV transmural scar, which may influence LV lead positioning Figure 1. Large-scale clinical trials are needed to evaluate the role of such modalities in predicting the long-term response to CRT [20–24]. 

## 3. 12 Leads ECG Remains the Gold Standard Test for CRT Patient Selection

Despite the wide availability of clinical and investigational imaging modalities to evaluate the patient response to CRT with variable accuracy, a simple 12-lead remains the standard test for patient selection. Several ECG parameters used to predict the response to CRT, including baseline rhythm, QRS duration, QRS morphology, LV activation sequence, and the PR interval.

### 3.1. QRS Duration

Prolonged QRS duration (≥120 ms) as measured on the standard 12-lead ECG is the most commonly used parameter in clinical practice to identify eligible candidates for CRT [25–28]. Despite the apparent simplicity and the reasonable reproducibility, accurate measurement of QRS duration remains a clinical challenge and an operator dependent. The main source of error seems to be in identifying the beginning and the end of QRS complex on surface ECG. The onset and the end of the QRS complex may be isoelectric, resulting in underestimation of the actual QRS duration. Other potential sources of error include fluctuation of the baseline and presence of a notch or a pacing spike at the onset of the QRS complex or contamination of the QRS complex by the repolarization changes. Computer measurements may provide more precise and more reproducible measurements in presence of a good quality 12-lead ECG [29]. 

### 3.2. Normal QRS Duration

More than 27% of heart failure patients with reduced left ventricular systolic function and QRS duration <120 ms have evidence of mechanical dyssynchrony by TDI, and the presence of which seems to be associated with increased mortality [30–32]. Few non-randomized studies suggested a beneficial outcome from CRT in this patient population; however, the RethinQ study showed no benefit in 172 patients with QRS duration <130 ms and mechanical dyssynchrony randomized to biventricular implantable cardioverter defibrillator against the control group. Furthermore, at six months there was no difference in Peak VO2, 6-minute walk test, LV reverse remodeling and quality of life score between the treatment and control groups [33–35]. 

### 3.3. Intermediate QRS Duration

The degree of QRS duration prolongation is an indicator of severity of electrical dyssynchrony [30]. QRS duration of 120 milliseconds or greater had been used as an entry criteria of major clinical trials (COMPANION, CARE-HF, RAFT, and REVERSE) [25–28]. Small studies using hemodynamics or peak oxygen consumption endpoints suggest that patients with intermediate QRS duration (QRS between 120 and 150 milliseconds) may not benefit from CRT [36, 37]. However, a meta-analysis that included the COMPANION, CARE-HF, REVERSE, MADIT-CRT, and RAFT trials found that CRT was effective in reducing adverse clinical events in patients with heart failure and a baseline QRS interval of 150 milliseconds or greater, but not in patients with a QRS of <150 milliseconds, and this difference in response between these QRS subgroups was seen in all New York Heart Association (NYHA) functional classes [38]. 

### 3.4. QRS Morphology

Baseline QRS morphology is probably equally important as QRS duration to predict response to CRT. Patients with a prolonged QRS duration may have a left bundle-branch block (LBBB), right bundle-branch Block (RBBB), nonspecific intraventricular conduction delay (IVCD), or paced rhythm. The presence of typical LBBB morphology is a strong predictor of response compared with right bundle branch block (RBBB) morphology and non-specific intraventricular conduction delay (IVCD) that has a much lower probability of CRT response [39, 40]. 

### 3.5. LBBB and LV Activation Patterns

In LBBB significant depolarization delay between the anteroseptal and posterolateral walls occurs which thought to explains the efficacy of CRT in this patients population. Careful evaluation of the QRS morphology in patients with apparent LBBB may yield important further information. An early report by Grant and Doge suggested that reversal of the intraventricular septal activation pattern should occur with the onset of LBBB, which is reflected in the initial 40 ms of the QRS complex; however, these expected changes were absent in 40% of the study patients who developed new LBBB [41]. Similarly Auricchio et al, using 3-dimensional (3D) nonfluoroscopic contact and noncontact mapping, studied the LV activation pattern (including LV endocardial breakthrough site, transseptal activation time, and duration of LV endocardial activation) and found that 32% of patients with apparent LBBB had <20 ms delay between the RV activation compared to LV endocardium and >40 ms in the remaining group, and the mean QRS duration was significantly different between the two groups (133 ± 28 ms, versus 170 ± 16 ms, resp.) [42]. Based on these observations and their own work Strauss and Sylvester argued that a QRS duration of 120–140 ms often represent left ventricular hypertrophy rather than a true LBBB and proposed that the criteria for complete LBBB should include QRS duration >140 ms in men or 130 ms in women. QS or rS in leads V1 and V2 and mid-QRS notching or slurring in at least two of leads V1, V2, V5, V6, I, and aVL [43].

**Figure 2:**
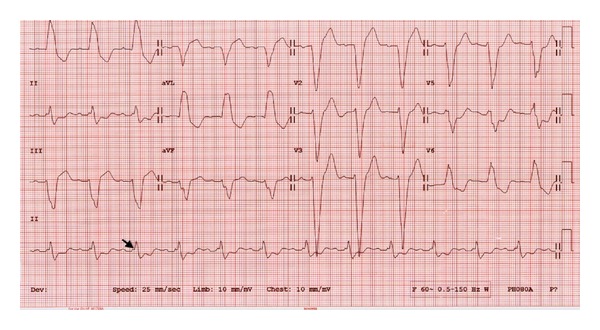
Baseline ECG from CRT super responder showing several predictors of good response including sinus rhythm, long PR interval, typical LBBB with mid-QRS slurring in lateral leads, QRS duration >200 ms, and long LVAT_max⁡_ measured by subtracting RVAT from the QRS duration. Arrow indicates the end of RVAT.

In a study of 202 consecutive heart failure patients with LBBB, Sweeney et al. developed a predictive model to test the hypothesis that the probability of reverse volumetric remodeling could be predicted by the ventricular activation pattern on the 12-lead ECG before and after CRT. Their main findings were that activation wave front fusion on the paced post-CRT ECG and prolonged maximum LV conduction time (LVAT_max⁡_) on baseline ECG are associated with higher probability of reverse remodeling. LVAT_max⁡_ is the difference between the total QRS duration and the right ventricular activation time (RVAT), where the RVAT represents the interval between the beginning of QRS and the early QRS notch (Figure 2) [44]. In the most recent ACCF/AHA/HRS guidelines update class I, indication for CRT was given only to symptomatic patients in sinus rhythm who have LBBB with a QRS duration greater than or equal to 150 ms and LV ejection fraction less than or equal to 35% [27].

### 3.6. RBBB and Nonspecific IVCD

Unlike LBBB, ventricular activation is not largely affected in RBBB, therefore from theoretical perspective CRT is not expected to be effective in this subgroup of patients [45]. Less than 15% of patients in the large controlled CRT trials had RBBB on baseline ECG, and as a result most available clinical data addressing the efficacy of CRT in RBBB are derived from retrospective data analyzing a relatively small number of patients [8, 9, 12, 13, 46]. Similarly, prospective studies included only a small number of patients with RBBB [47]. Systematic review of five studies which reported data on patients with RBBB including 259 patients randomized to CRT and 226 randomized to non-CRT showed unfavorable outcomes in patients with CRT [48]. Recently a meta-analysis of 5356 patients included in the major CRT trials, COMPANION, CARE-HF, MADIT-CRT, and RAFT trial, showed no benefit from CRT in patients with RBBB (RR: 0.91; 95% CI: 0.69–1.20; *P* = 0.49) or nonspecific IVCD (RR: 1.19; 95% CI: 0.87–1.63; *P* = 0.28) [40]. Furthermore, there was no heterogeneity among the clinical trials in the lack of benefit in non-LBBB patients. The benefit of CRT is significantly higher in LBBB compared with non-LBBB group; *P* = 0.0001 [40].

### 3.7. Patient Rhythm, P Wave Morphology and the PR Interval

Patient rhythm, interatrial conduction delay and the magnitude of atrioventricular delay, as represented by the native PR interval are additional valuable information that may influence CRT response and can be easily obtained from the baseline 12-lead ECG. 

The role of CRT in patients with atrial fibrillation is not well established: major clinical trials of resynchronization included mainly patients in sinus rhythm. However, other studies suggested a positive outcome in AF patients [49–51]. A meta-analysis of 1,164 patients in five studies showed that patients in AF had a significant improvement after CRT, with similar or improved ejection fraction as sinus rhythm patients, but the functional improvement was less [52]. 

Interatrial conduction delay is characterized by a wide and notched P wave in lead II with a wide terminal negative deflection in lead V1. Significant interatrial delay may results in left atrial contraction during LV systole, which may negatively affect CRT outcome. In such cases simultaneous activation of both atria could be achieved by implantation of the atrial lead in the interatrial septum [53]. 

To ensure near 100% biventricular pacing in CRT, the programmed AV delay should be shorter than the native PR interval, this programming may truncate the left ventricular filling resulting in a suboptimal response to CRT; however, the presence of a long native PR interval may permit a more physiological AV delay programming. Subgroup analysis of patients in the COMPANION trial demonstrated that randomization to CRT was associated with a reduction in the endpoint, but the strength of the association was greater for those with prolonged PR (hazard ratio = 0.54; *P* < 0.01) versus normal PR (hazard ratio = 0.71; *P* = 0.02) intervals [54].

## 4. Conclusion

Prediction of CRT response is a complex and subject of extensive research over the past decade. Despite all we know about CRT, a significant proportion of heart failure patient dose not respond to CRT. However, careful analysis of simple 12-lead ECG can yield impressive data difficult to replace by any of the available more sophisticated clinical tools.
